# Developing a Health Care Transition Intervention With Young People With Spinal Cord Injuries: Co-design Approach

**DOI:** 10.2196/38616

**Published:** 2022-07-28

**Authors:** Emily Alice Bray, Bronwyn Everett, Ajesh George, Yenna Salamonson, Lucie M Ramjan

**Affiliations:** 1 School of Nursing and Midwifery Western Sydney University Penrith Australia; 2 Ingham Institute Applied Medical Research Liverpool Australia; 3 School of Dentistry Faculty of Medicine and Health University of Sydney Camperdown Australia; 4 School of Nursing Faculty of Science, Medicine and Health University of Wollongong Wollongong Australia

**Keywords:** co-design, participatory action research, health care transition, pediatric health care, adult health care, spinal cord injury

## Abstract

**Background:**

Successful transition from pediatric to adult health care settings supports long-term health management and better overall outcomes in all domains. However, young people with spinal cord injuries (SCIs) continue to report challenges and unmet needs during the transition process. Including end users in health care research and intervention design is paramount as interventions designed in this way better meet their specific needs and are often more innovative. Although studies have reported involving young people with chronic conditions in the development of health care transition (HCT) interventions, few details have been provided as to how this was achieved.

**Objective:**

This study outlined the co-design and development of an HCT intervention to support young people with SCIs. It contextualized the co-design process, methods, materials used, and steps implemented from defining the problem to conceiving and designing the solution. This was accomplished by understanding and listening to end users’ needs and recommendations for HCT.

**Methods:**

Using participatory methods, this qualitative study reports the co-design of an HCT intervention to support young people with SCIs and parents or caregivers. Two co-design workshops were conducted: one with young people with SCIs and one with parents and caregivers. Categories were defined through a hybrid deductive and inductive qualitative content analysis process that was informed by the Care Transitions Framework and guided the development of the HCT intervention. Following the creation of a prototype intervention, young people with SCIs, parents and caregivers, and key pediatric SCI stakeholders provided feedback on the intervention content and design in focus groups. Similar to the workshops, the focus groups were analyzed using a hybrid deductive and inductive qualitative content analysis process informed by the Care Transitions Framework. The Enhancing the Quality and Transparency of Health Research guidelines for qualitative research (Consolidated Criteria for Reporting Qualitative Research) were applied.

**Results:**

Overall, 4 young people and 4 parents or caregivers participated in the co-design workshops. Key recommendations for the HCT intervention were that participants wanted a “one-stop shop” for all their transition information needs and an editable portable medical summary to take with them to appointments. On the basis of the analysis of participants’ recommendations from the workshops, it was determined that a website would be an appropriate hosting platform for the interventions. The focus group feedback on the design and content of the prototype website was extremely positive, with minor recommendations for improvement.

**Conclusions:**

This is the first study to co-design and develop an HCT intervention in partnership with young people with SCIs and parents and caregivers. Although the study sample was small, it has shown that it is possible to meaningfully engage and empower young people with SCIs and parents and caregivers in the co-design of an HCT intervention.

**International Registered Report Identifier (IRRID):**

RR2-10.1136/bmjopen-2021-053212

## Introduction

### Background

A key goal in the rehabilitation of children with a spinal cord injury (SCI) is to facilitate the attainment of a productive and satisfying life by addressing developmental milestones and the provision of education on managing the complex health issues that arise because of aging and living with an SCI [1]. Importantly, a seamless transition from the pediatric to the adult health care systems, termed health care transition (HCT), is a significant and critical factor in supporting the fulfillment of goals while fostering independence and improved health outcomes [2].

Supporting a seamless HCT has been on the international health care agenda for >3 decades [3-5], yet young people with chronic conditions continue to report facing a multitude of barriers to their move [6,7]. These barriers include fear of losing established relationships with pediatric providers and forming new ones with adult providers, inadequate preparation for and information on the adult health care system, lack of self-management skills and disease knowledge, and poor communication between the pediatric and adult health care providers [6,7]. Consequently, these barriers result in poor health outcomes such as nonadherence to treatment and medication, loss to follow-up, increased hospital admissions, and patient dissatisfaction [7]. As such, there is an opportunity to reduce the difficulties faced by young people with chronic conditions through the development of HCT interventions that prepare them for the move and improve the transfer process.

In recent years, there has been significant progress made toward improving HCT for young people with chronic conditions. Evidence suggests that a structured HCT process, including planning for transition, transfer assistance, and integration into adult services, can improve outcomes for young people with chronic conditions, such as patient satisfaction, population health, and the use of health care services [2]. However, to date, HCT research has been disease-specific, and studies are typically characterized at the lowest evidence level, making them difficult to apply in various contexts [2,8].

Similar to other young people with chronic conditions, young people with SCIs face many barriers and facilitators in their transition to adulthood with regard to both health care services and normative life milestones such as education, employment, social participation, and independent living [9]. A study conducted in the United States on 23 young people with SCIs and their caregivers identified processes within health care that acted as both a barrier to and facilitator of the transition to adulthood [9]. Facilitators included health care support comprising the transfer of medical records, clear communication of transition timelines and expectations, referrals to adult services, and collaboration between the pediatric and adult settings. Health care barriers to transition included complex adult services, limited resources, and minimal previous exposure to the adult health care setting. In particular, the study indicated that, when it came to health care, there was a need for more condition-specific education for local, nonspecialized health care providers; better communication among health care providers; and an accessible, concise, and comprehensive medical history [9]. Evidence on the availability of HCT interventions for young people with SCIs that attempt to address these transition needs is scarce, with only 1 Australian article offering an explanation of their HCT efforts [10].

As the importance of end-user involvement in health care research and intervention design has been increasingly recognized, there has been a shift away from end users being passive participants in research, where research is conducted on them, to active participation, where research is conducted with them [11,12]. One such research approach, participatory action research (PAR), is particularly useful in co-design. PAR involves researchers collaborating with service users and key stakeholders in a collective and reflective inquiry to understand and improve practices and situations [13]. This process acknowledges that participants have knowledge and expertise to share, which is particularly important for the disability community whose voices have too often been silenced [14].

Collaboration within the PAR process takes place through iterative cycles of “planning, acting, and review” [15], and co-design can be used to facilitate the action stages. The co-design process actively involves all stakeholders in identifying solutions to local problems using their experience and expertise to explore the current needs of service users and develop and test concepts before improving the prototype in an iterative process [12]. Interventions designed in this way better meet the specific needs of end users and are often more innovative [11].

Although studies have reported involving young people with chronic conditions in the development of HCT interventions, few details have been provided as to how this was achieved [16]. As such, more transparency is needed regarding the process, methods and materials used to include young people with chronic conditions in intervention development.

The aim of this qualitative study was to fill this knowledge gap by providing a detailed explanation of the process involved in understanding the needs and recommendations for HCT as part of the co-design and development of an HCT intervention with young people with SCIs. This is the first study to co-design and develop an HCT intervention in partnership with young people with SCIs and parents or caregivers. Please note hereafter and unless otherwise specified, the term caregivers will be used to denote parents or caregivers. Addressing the needs of individuals and the current gap in services has the potential to improve transition outcomes and the quality of life of children and young people with an SCI.

### Research Context and Conceptual Framework

The work presented in this paper forms part of a wider 3-year study informed by a PAR approach that aimed to co-design, develop, implement, and evaluate an HCT intervention to support young people with SCIs in New South Wales (NSW), Australia. Further details on the current SCI services in NSW can be found in an evidence series by the Agency for Clinical Innovation [17].

The study protocol has been published elsewhere [18]. In summary, the 3 study phases were informed by the Care Transitions Framework [19]. The Care Transitions Framework is an adaptation of the Consolidated Framework for Implementation Research, an established conceptual framework in implementation science [19]. The framework guides the research and evaluation of care transition interventions within a variety of settings and can be used in parts or as a whole. Organized into 8 domains, the Care Transitions Framework provides a comprehensive guide to potential questions that can be explored depending on the nature of the research and its goals [19]. Five of the domains for the overall study are presented in Multimedia Appendix 1. The 3 remaining domains (external context, organizational characteristics, and characteristics and roles of providers) were addressed as part of the prestudy consultation process.

## Methods

### Study Design

The overarching 3-year PAR study consisted of 3 phases (Multimedia Appendix 1). Phase 1 used semistructured interviews to explore the experiences with HCT of young people with SCIs and caregivers and has been published elsewhere (Bray et al, under review). Briefly, these interviews revealed that young people with SCIs and caregivers faced barriers and had unmet needs in their transition to adult health care services (Bray et al, under review). During the phase 1 interviews, young people with SCIs and caregivers identified the need for a coordinated and streamlined handover from pediatric to adult health care providers and a “one-stop shop” for transition information, such as how it occurs, who to call for ongoing support and advice, and tips on how to transition successfully (Bray et al, under review).

This paper presents the findings from phase 2 of the PAR study, which consisted of 2 parts. Phase 2a built on the findings of the phase 1 interviews and engaged both young people with SCIs and caregivers as individuals with “lived experience” in co-design workshops to inform the development of a prototype HCT intervention. In continuation of the co-design process, phase 2b used focus groups to gather feedback on the prototype HCT intervention, leading to further refinement before phase 3 evaluation. As part of the PAR approach, the participatory methods used included collaboration, active engagement, and reflection that occurred through iterative cycles of “planning, acting, and review” [13,15]. This phase (phase 2) of the PAR study formed part of the acting phase of PAR and supported working collaboratively with the community of interest to identify actions necessary to achieve the desired outcomes.

This study has followed the Standards for Reporting Qualitative Research [20].

### Ethics Approval

This study received ethics approval from the Western Sydney University Human Research and Ethics Committee (H14029) and was registered with the Australian New Zealand Clinical Trials Registry (ACTRN12621000500853). Participation in this study was voluntary. Written consent was obtained at the beginning of the study, and verbal consent was obtained at the beginning of each workshop or focus group. All young people aged <16 years also required written consent from a caregiver. Transcripts were deidentified, and participants were assigned a pseudonym to maintain confidentiality. Participants received an Aus $30 (US $20.30) e–gift card each as a *thank you* for their time.

### Participants

Owing to the limited number of pediatric-onset SCI cases in Australia [21,22], participants were recruited from both metropolitan and rural areas of NSW. To be eligible for inclusion, participants needed to be young persons aged between 14 and 25 years and have sustained a pediatric-onset traumatic or nontraumatic SCI (at or before the age of 16 years) or be a parent or caregiver of the same. Individuals had to be preparing for or have made the transition from pediatric to adult health care services (including tertiary hospitals and community services). Sufficient English-language proficiency was another requirement to ensure that all participants could fully engage in the conversation. Exclusion criteria were individuals who were receiving rehabilitation treatment for an injury sustained in the previous 12 months. This exclusion criterion was implemented so as not to burden the individual or their family with the demands of participating in research or risk causing any additional emotional distress during this tremendous period of adjustment. Individuals with neural tube defects such as spina bifida were also excluded. Although the authors acknowledge that individuals with neural tube defects share many of the same clinical characteristics and complications as those with SCIs, these individuals also demonstrate distinct features [23]. Research on HCT also typically focuses purely on SCIs [9] or spina bifida [24,25], with children and young people with spina bifida reported as a separate group supported by their own services. As such, this study focused specifically on young people with pediatric-onset traumatic or nontraumatic SCIs.

### Recruitment

Young people with SCIs and caregivers were recruited from the individuals who had participated in the previous phase of the overall study. We contacted all 9 participants first via email to determine availability. We then contacted the participants by phone to confirm their availability and book the workshop or focus group. Each participant was sent an SMS text message the day before the workshop or focus group to confirm attendance.

### Rigor and Reflexivity

To maintain rigor within the study, credibility was ensured by reporting verbatim excerpts, tracking coding and category decisions, and confirming these through researcher triangulation [26,27]. Although the study findings and the tailored intervention may not be generalizable to populations outside the study setting, providing a comprehensive description of the co-design process through detailed reports, thick descriptions, and analysis of contextual details, as described by Ponterotto [28], may allow for the transferability of the research method across contexts and medical conditions [29]. A comprehensive commentary reflecting on and cataloging the progress, obstacles, and successes of the research process increased dependability and confirmability by providing an audit trail for the study [29].

The primary researcher in this study (EAB) was an individual with an SCI, and a crucial step in their personal reflexivity [30] involved reflecting on how their position and perspective affected the study, in particular how being a member of the SCI community and having shared experiences but also common contacts allowed for the development of relationships with young people and caregivers. Developing these relationships at the beginning of the study and allowing time for non–research-focused conversations created a safe space in which young people could express their needs. Nevertheless, as the researcher sustained their SCI at the age of 22 years and did not use any pediatric services, the young people were the experts in this area.

## Co-design Workshops (Phase 2a)

### Methods

#### Overview

Two workshops were run on the web (owing to the COVID-19 pandemic) via videoconference (Zoom Video Communications): one for young people with SCIs and one for caregivers. Two researchers facilitated the workshops, one taking on the role of lead facilitator (EAB) and the second acting as cofacilitator and note-taker (LMR). Each workshop was run as a single group (all participants together all the time), with each participant invited to contribute their thoughts during each activity. Discussion prompters (eg, Ideaflip [Biggerflip Ltd] and Microsoft PowerPoint) were used in the workshops to organize material from discussions, enhance feedback, and guide intervention development. The workshops were recorded and transcribed with the participants’ permission.

#### Preworkshop Preparation

A reference group was consulted to provide expert advice on the appropriateness of the co-design workshop activities, identify any issues or barriers that could impede the success of the workshop, and provide advice on how to resolve these issues or barriers. This reference group consisted of a young person with an SCI and 3 health care professionals (n=1, 33% clinical nurse consultants and n=2, 67% occupational therapists) from 3 different pediatric SCI health care service providers.

Approximately 1 week before the workshops, the facilitators met to clarify roles and review the workshop schedule and timing of activities. Participants were also sent an information booklet (Multimedia Appendices 2 and 3) and a link to the videoconference meeting by email.

#### Workshop Warm-ups

Each workshop began with a reminder of the aims of the study followed by a fun icebreaker activity (Multimedia Appendix 4) to help build rapport and ease any anxiety. Given that we were discussing issues relating to their personal experience transitioning between health care services, we asked that participants maintain confidentiality, especially if any sensitive issues were discussed. At the same time, each participant was encouraged to share as much or as little as they were comfortable with. This created an atmosphere that promoted open and honest communication and sharing. In the young persons’ workshop, this information was reiterated at the start of the second activity (without the icebreaker) as some young people joined the workshop after the completion of the first activity.

#### Young Persons’ Co-design Workshop

**Figure 1:**
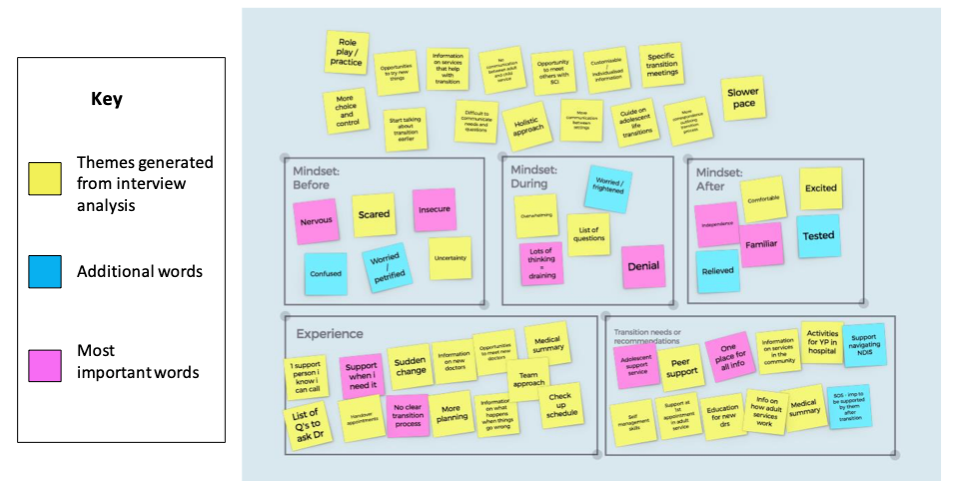
Screenshot of the first young peoples’ co-design workshop activity.

The young persons’ workshop was 120 minutes long and consisted of 2 activities. The first activity required participants to review the thematic analysis of the semistructured interviews conducted by EAB and LMR as part of phase 1 of the research study. Participants were encouraged to reflect on some of the common ideas and concepts (codes) generated by the facilitators, confirm their authenticity, and compare and discuss analytical decisions as they grouped codes into themes. A web tool (Ideaflip) was used to create a whiteboard with Post-it notes to organize each idea or concept into predetermined boxes titled “Mindset: before,” “Mindset: during,” “Mindset: after,” “Experience,” and “Transition needs or recommendations” (Figure 1). These predetermined box labels aligned with the “Characteristics and Roles of Patients and Caregivers” domain of the Care Transitions Framework. Participants were also asked to add words or ideas that they believed had been omitted and highlight the most important word for them in each box. Participants’ reflections on common ideas, concepts, and themes confirmed the thematic analysis conducted by EAB and LMR and have been published elsewhere (Bray et al, under review).

For the second activity, the researchers used the future workshop method [31,32] to facilitate discussion and generate ideas for the development of the HCT intervention. The future workshop method consists of 3 phases: a critique phase, a fantasy phase, and an implementation phase [31,32]. In the critique phase, the participants were asked to identify deficits or challenges related to the HCT experienced by young people with SCIs. The questions posed included the following: “What is the change you want to see? Or what did you need most to support your move but didn’t have or receive?” In the fantasy phase, the participants were asked the following: “In a perfect world, how can this be achieved? Or what could we develop to support your move?” In the implementation phase, the participants transformed the “perfect world” ideas into a design for a practical and realizable HCT intervention (“How can we make this possible today?”). Participants had been provided with these 3 questions before the workshop in their information booklet. Using a Microsoft PowerPoint slide as a whiteboard, we discussed the participants’ answers to the 3 posed questions and asked them to discuss their thought processes and ideas (Multimedia Appendix 5). The participants built on each other’s ideas.

#### Caregivers’ Co-design Workshop

This 1-hour workshop mirrored activity 2 of the young persons’ workshop (Multimedia Appendix 6). The questions posed to the caregivers included the following: (1) “What is the change you want to see? Or what did you need most to support your child’s move but didn’t have or receive?” (2) “In a perfect world how can this be achieved? Or what could we develop to support your child’s move?” (3) “How can we make this possible today?”

#### Workshop Evaluation

At the end of the young persons’ workshop, a short evaluation of the co-design workshop process took place. A web tool called Mentimeter (Mentimeter AB) was used to pose the following question: “Using 5 or more words, describe how you felt as a participant and what you thought of the workshop?”

#### Workshop Follow-up

Following the workshops, participants were provided with a link to a secure web-based Google document that included the questions posed in the workshops and a summary of the topics and ideas discussed. Participants were encouraged to cross-check information, add any information not covered during the workshops, and continue to develop the ideas generated.

#### Workshop Analysis

The audio recordings were transcribed and used in conjunction with the discussion prompters (Microsoft PowerPoint) to generate content themes for guiding the development of the HCT intervention. The researchers used a hybrid approach of deductive and inductive qualitative content analysis [26] of the transcripts, notes, and materials produced after each workshop. The reason this analysis method was chosen was that it allowed the researchers to organize and understand the data in a meaningful way and, through a manifest analysis, allowed the researchers to describe “*what* the informants actually say” by remaining close to the verbatim text [33]. The analysis was conducted in 3 stages: preparation, organization, and reporting [26]. In the first stage, the researchers immersed themselves in the data to obtain a sense of them as a whole. In the next stage, the researchers organized and condensed the data into meaning units through a process of open coding, categorization, and abstraction for the inductive approach and used the Care Transitions Framework to develop a categorization matrix for coding for the deductive approach (Multimedia Appendix 7). Finally, the contents of the categories and subcategories are described in detail as part of reporting the results.

### Results

#### Participants

Young people joined and left the workshop at different times because of other commitments. In total, 2 participants, both female with tetraplegia aged 20 and 21 years, were present for the first activity of the young persons’ workshop. A total of 3 participants, 2 (67%) female (n=1, 50% with tetraplegia and n=1, 50% with paraplegia) and 1 (33%) male with tetraplegia aged between 17 and 20 years were present for the second activity; 1 (33%) had attended the previous activity, and 2 (67%) new people joined for the second activity. In total, 4 participants, all mothers, attended the caregivers’ workshop. A total of 75% (3/4) of the mothers had children with tetraplegia, and 25% (1/4) had a child with paraplegia. Only 1 young person and 1 caregiver had not yet transitioned. In total, 89% (8/9) of the original participants from phase 1 of the overall PAR study contributed to the co-design workshops.

#### Recommendations for the HCT Intervention

##### Overview

Data from the workshops were categorized in alignment with the Care Transitions Framework as summarized in Table 1.

**Table 1:** Workshop analysis content categories.

Care Transitions Framework domain and category		Subcategory
**Intervention characteristics**		
	What is the intervention designed to achieve?	Coordinated handover between services: “For there to be more of a relationship built with the doctor before the transition occurs.” Greater independence: “I want to start moving that stuff to her, getting her to do things independently.” Peer connection: “Support group of the people going through the same thing as you.”
	What are the features of the intervention?	Information on the transition process and adult health care system: “Written information and summary on the transition process.” Medical summary and contact list: “Parallel lists of what was before and what was now. Like, pediatric versus adult.” One-stop shop for resources: “There should be pamphlets or a website with all the information.” Support to connect with others: “To be doing something together to help form relationships.”
	Who is the intended target group?	—^a^

^a^No subcategory.

##### Category 1: What Is the Intervention Designed to Achieve?

Although caregivers wanted an intervention that supported their children in achieving greater independence, young people wanted the intervention to offer a space for peer connection. However, both caregivers and young people requested that the intervention support a coordinated handover between the pediatric and adult health care services and their multidisciplinary team members, including medical, nursing, rehabilitation, and allied health professionals.

##### 1.1: Coordinated Handover Between Services: “For There to Be More of a Relationship Built With the Doctor Before the Transition Occurs”

Both young people and caregivers advocated for a smoother and more streamlined HCT. They believed that greater communication between the 2 health care settings (pediatric and adult) was essential for this to happen:

The adult service neurologist knew her paediatric neurologist and that is [a] huge help because they can chat between themselves, especially [because] we have the same neurologist for 15 years and he knew everything about Drew. It’s so easy [for them] to communicate, rather than going through me.

Morgan, caregiver

In addition, participants wanted their pediatric health care team to organize the initial introductions of the family unit to the new adult health care team before transition:

I think just having an introduction as a family transitions across would be good.

Jude, caregiver

They felt this would reduce the need for young people and their caregivers to have to repeat their stories to different health care professionals:

Just a handover so people know where you are and you’re not having to repeat yourself all the time.

Kris, caregiver

Furthermore, young people indicated a belief “that some doctors underestimate how big the transition actually is for young people*”* (Jamie, young person) and, as such, more support should be provided during the transition process. This would allow young people and their caregivers to build a relationship and rapport with their new health care team before moving and, in so doing, it would ease any anxiety around the transition:

For there to be more of a relationship built with the doctor before the transition occurs...so that the doctors are more aware of how the doctors in the children’s hospital provide support and possibly adopt that, even though they’re in the adult hospital.

Jamie, young person

##### 1.2: Greater Independence: “I Want to Start Moving That Stuff to Her, Getting Her to Do Things Independently”

Caregivers acknowledged that, around the age of 16 years, young people legally could take charge of their own health care but that a lack of self-management skills at this age can result in unanticipated errors that can both be financially costly and have a detrimental impact on one’s health:

The issue of being 16 [is that] Taylor was in charge, really on paper—he’s in charge of his own health care, while he wanted us, as parents, to guide some of that. He wanted to make his own decisions and [has] every right to...Taylor is the voice of his own body. He’s not always right, but it’s 16 when he’s making some of those choices and those choices are very expensive choices if you’re buying a chair or you’re paying for things...there’s so many things that we’ve got wrong that have been a trial and error.

Rory, caregiver

Further to their lack of skills and knowledge, some caregivers identified that their child did not want to take charge of their own health care. However, caregivers saw the relinquishing of responsibility for health care decisions to the young person as an important milestone in the move to adult health care services:

She doesn’t even want to entertain the thought of ordering products for herself or ringing up to get a new commode pusher. She wants me to do all that for her, I’m happy to, but I would like to see that, in the transition coming up in the next couple of years, I want to start moving that stuff to her, getting her to do things independently herself.

Kris, caregiver

Going forward in the young persons’ move to adult health care services, caregivers saw that a requirement of the HCT intervention should be to build independence and the skills they need to manage their health care on their own:

So there’s definitely something for me about...this kind of thing of letting young people become young people with that fierce independence, but to develop the skills that they actually see a chair as a piece of equipment that’s necessary, that they see that they need that kind of life skills of being able to manage in situations.

Rory, caregiver

##### 1.3: Peer Connection: “Support Group of the People Going Through the Same Thing as You“

All young people identified having an intervention that supported the opportunity to connect with others going through a similar experience as an important priority:

A support system where everyone going through the same experiences is able to kind of bond and start friendships.

Jamie, young person

##### Category 2: What Are the Features of the Intervention?

Young people and caregivers clearly outlined what they imagined an HCT intervention would need to include to achieve the desired outcomes outlined in the previous sections. These included information on the transition process and adult health care system, a medical summary including a contact list for health care professionals, accessible resources all located in one place, and a space to connect with others.

##### 2.1: Information on the Transition Process and Adult Health Care System: “Written Information and Summary on the Transition Process”

Part of the struggle participants faced in their transition was a lack of knowledge of how and when the transition to adult health care would occur as well as how the adult health care system works:

It seems that some services change over or changed over when Jessie turned 16 and then others have stayed with paediatric services and so we’ve got this mix of some adult doctors ongoing with Jessie and some who are still in the paediatric system and it seems very messy for us. We’re not sure whether we’re in the adult system yet or in the children’s system.

Jude, caregiver

I didn’t have the knowledge. For example, in intensive care in paediatrics, we always had the same person and he was constant. Now, when we ended up in ICU for six weeks, every four days is a new doctor and that is so disturbing. Having 15 years [with] the same doctor and now, every four days, it was really hard to follow and it’s not easy.

Morgan, caregiver

To address this knowledge gap, participants recommended having more information and education on the transition process and the adult health care system:

Written information and summary on the transition process.

Drew, young person

##### 2.2: Medical Summary and Contact List: “Parallel Lists of What Was Before and What Was Now. Like, Paediatric Versus Adult.”

Participants identified that a coordinated handover could be further supported with written documentation that is readily available to health care professionals, young people, and caregivers. A medical summary specifically created for the handover would mean that young people and their caregivers would not have to retell their stories:

A handover so people know where you are and you’re not having to repeat yourself all the time. An updated information folio somewhere where you can access your information and where the doctors can access your information.

Kris, caregiver

Participants envisioned that the medical summary would include the young person’s medical history, a schedule for annual appointments and scans, experiences they valued in the pediatric health care setting, and accomplishments:

The medical history of each person, what experiences from the paediatrics that they really enjoyed and would love to be integrated in the adult hospitals just to make it not as daunting when the transition occurs.

Jamie, young person

Getting a clear list of what are the ongoing check ins that Jessie needs for bladder, for bowel, for bones and how frequently we have to have those scans and tests done. I’m still trying to piece that together and it’s incredibly messy and I keep thinking I’m going to miss him having an important scan or test done because I don’t have a schedule that says “every two years, Jessie will need a bone scan. Every 12 months, he’ll need a kidney test.”

Jude, caregiver

Only 1 young person in the group described having a summary of their health care created for the handover process. However, despite it being given to the new health care team, Taylor felt that he had to repeat this information and supplement it. He also reported that he thought he probably had a copy of that summary but was not sure where it was now:

I think it had that, but I had to also repeat what could have been written down more than added to the summary.

Taylor, young person

In addition to the transition-specific medical summary, participants requested a contact list for their health care team that clearly displayed the pediatric health care professional and who would be taking over that role in the adult health care setting along with their contact details:

It would be nice to have the parallel lists of what was before and what was now. Like, paediatric versus adult...First [the] name of every condition, then name of every doctor or CNC [Clinical Nurse Consultant] and then equivalent or similar match in adult services and their contacts.

Morgan, caregiver

##### 2.3: One-stop Shop of Resources: “There Should Be Pamphlets or a Website With All the Information”

What was evident from the discussions was that participants desired a “one-stop shop” or a repository where all HCT information was packaged in an easy-to-understand format and accessible:

There should be pamphlets or a website with all the information that people need to make it easily accessible in one place.

Jamie, young person

It's a package of education, health and everything else that's in our lives that can't be segregated.

Rory, caregiver

When asked about the information and resources they would like to be included in an HCT intervention, there were several requests (Table 2).

Young people also spoke about having the opportunity to share resources and collaborate, possibly using a web-based forum:

Just coming back to the opportunity to share the resources online, also possibly starting a forum within that in order to share—to help share those resources a bit more easily.

Jamie, young person

Finally, participants wanted information and resources to come from reputable sources such as physicians:

A list of resources provided by doctors possibly on the forum.

Jamie, young person

**Table 2:** Information and resources requested for inclusion in the health care transition intervention.

Information and resources requested	Participant quotes
Information on disability (general and SCI^a^-specific)	“Things to do with disability and all stuff.” [Ashley, young person]
Information on the difference between the pediatric and adult health care settings	“How the adult system works and possibly differentiates from the children’s system.” [Jamie, young person] “Someone to explain what’s the difference between paediatric and adult.” [Drew, young person]
Information on social activities	“Information on sport. Like wheelchair sports and disabled sports.” [Taylor, young person]
Alternate funding options	“For people who don’t get funding, like charities. Stuff like that to get equipment, wheelchairs, and stuff.” [Taylor, young person]
Tips on building self-management skills	“So managing all of these things is actually a skill which can be learnt either in some workshops or self‑education...maybe you can request from a...Social Worker who might come and show some tips to your child and some mind mapping or Excel spreadsheets.” [Morgan, caregiver]
Education and employment support	“It’s not just the transition of his care and things. It’s that transition to what you do beyond school and how do you do that when you have a spinal cord injury?” [Jude, caregiver]

^a^SCI: spinal cord injury.

##### 2.4: Support to Connect With Others: “To Be Doing Something Together to Help Form Relationships”

When asked to expand on how they would like to connect with others going through a similar experience, young people recommended having both one-on-one and group support options. They recommended that the one-on-one support be a formal program tailored to the young person’s individual needs, matching them with someone who is either of a similar age or injury or who may have experience of transition, depending on what the young person desires:

Maybe the same age, but also similar injuries. So if it’s someone who is able to walk, then someone who—I guess [a] support worker who may be able to walk.

Taylor, young person

The group support would be more informal and would involve monthly catch-ups of a more social nature (bowling, trivia, and games on the web):

Do an activity during that, instead of talking. To be doing something together to help form relationships.

Jamie young person

##### Category 3: Who Is the Intended Target Group?

The ultimate beneficiaries of the intervention are young people with SCIs. However, participants noted that they saw the intervention “as a package that everybody takes a role [in]*”* (Rory, caregiver)*.* As such, the HCT intervention needed to support young people, their caregivers, and health care professionals.

#### Workshop Evaluation

A total of 75% (3/4) of the young people completed the evaluation at the end of the co-design workshop answering the following question: “Using 5 or more words, describe how you feel as a participant today and what you thought of the workshop?” The feedback was positive, and young people reported that they felt valued and listened to and that the workshops were fun and informative (Multimedia Appendix 8).

## The Prototype HCT Intervention

**Figure 2:**
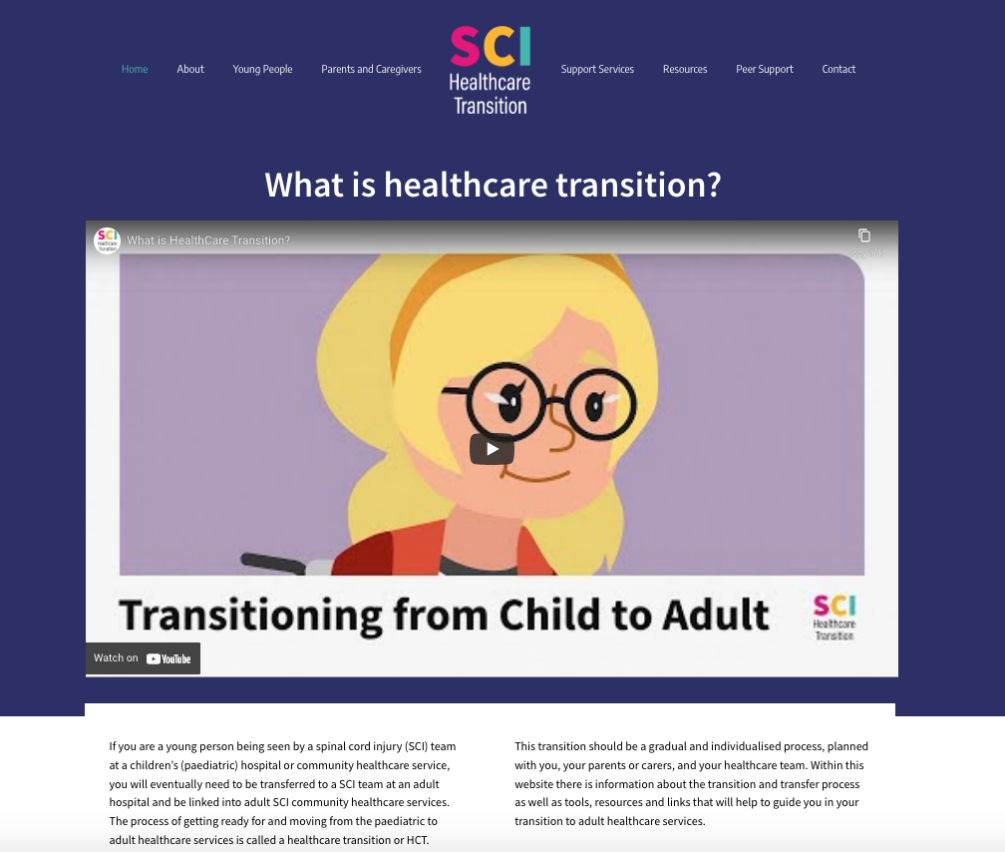
Screenshot of the SCI Healthcare Transition website home page. SCI: spinal cord injury.

On the basis of the recommendations from participants in the workshops, it was determined that a website would be an appropriate platform on which to deliver the suite of information needs. Content categories from the workshop were used to guide the development of the prototype HCT intervention, the SCI Healthcare Transition website [34] (Figure 2). The development of the website occurred over a period of 4 months and was supported by a web designer, motion graphics designer and video editor.

**Figure 3:**
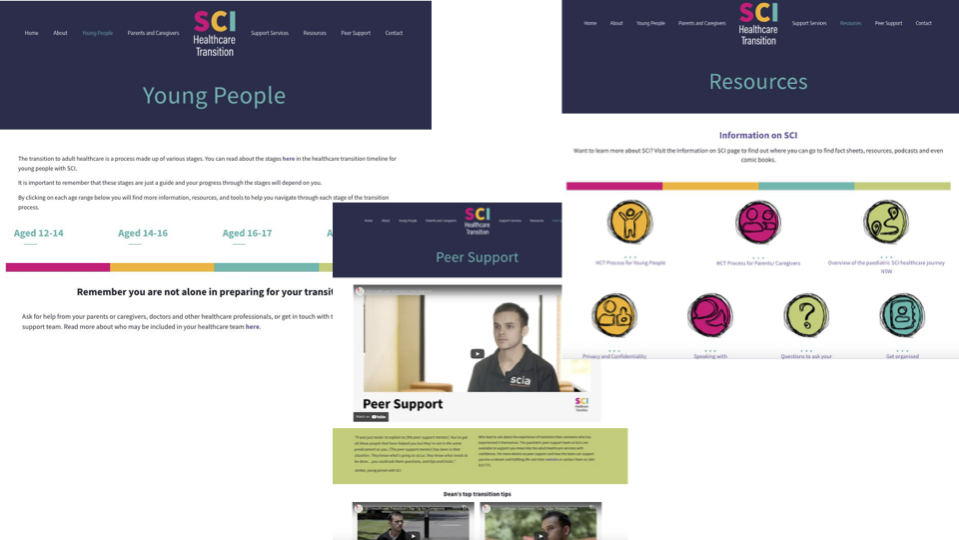
A total of 3 screenshots of the SCI Healthcare Transition website. SCI: spinal cord injury.

The website was designed with the aim of supporting young people with SCIs to achieve greater health care independence, support the smooth and coordinated handover from children’s to adult health care services, and offer young people with SCIs a way to access peer support. It aimed to achieve this by providing young people with SCIs and caregivers with a step-by-step guide to HCT categorized by age that included tools (eg, a goal-planning worksheet), tips (eg, a PDF on tips for talking to health care professionals), and resources (eg, information on SCIs) to help prepare for the move. It also provided a directory on where to access further support from health care professionals and peer mentors. The website used a combination of eye-catching colors and graphics along with videos, interactive quizzes, and PDFs that are both downloadable and can be filled in, with the intention of appealing to young people (Figure 3).

## Focus Group Evaluation of the Prototype HCT Intervention Development (Phase 2b)

### Methods

#### Overview

Similar to the process outlined for phase 2a, 2 focus groups were run on the web (owing to the COVID-19 pandemic) via videoconference (Zoom): one with young people with SCIs and caregivers and the other with the study’s reference group of SCI health care professionals. The proposed HCT intervention was presented to the 2 groups, each of whom provided constructive feedback on the overall content and the layout and structure, allowing for further refinement of the intervention. The focus group was facilitated by 2 of the study’s researchers, one taking on the role of lead facilitator (EAB) and the second acting as cofacilitator and note-taker (LMR). The focus groups were run as a single group (all participants together all the time). Similar to the workshops, discussion prompters (Microsoft PowerPoint) were used to guide the discussion and feedback. The focus groups were recorded and transcribed with the permission of the participants.

#### Co-design Process Evaluation

At the end of the focus group with young persons with SCIs and caregivers, a short evaluation of the co-design process took place. The researcher reflected on the co-design process with participants and gathered evaluative feedback.

#### Focus Group Analysis

As in phase 2a, the focus group transcriptions were used in conjunction with the discussion prompters (Microsoft PowerPoint) to develop content themes for guiding the refinement of the prototype HCT intervention.

### Results

#### Participants

Of the 9 participants contacted from phase 1 of the overall PAR study, 4 (44%) provided feedback to the focus groups. In total, 3 participants attended the young people with SCIs and caregiver focus group: 1 (33%) young person (female, aged 21 years with tetraplegia) and 2 (67%) caregivers (both mothers of children with tetraplegia). A caregiver (mother of a child with paraplegia) was unable to attend the focus group but submitted written responses to the questions.

A total of 3 participants were invited, and all attended the SCI health care professionals’ focus group. The health care workers were from 3 different pediatric SCI service providers, and each had different professions (clinical nurse consultant, occupational therapist, and physiotherapist).

#### Recommendations

##### Overview

Data from the focus groups were categorized in alignment with the Care Transitions Framework as summarized in Table 3.

**Table 3:** Focus group analysis content categories.

Care Transition Framework domain and category		Subcategory
**Intervention characteristics**		
	Does the website achieve what it was designed to achieve?	—^a^
	Website features	Successful features: “That was...really awesome” Recommendations for improvement: “One thing I thought would have been really useful is...”
**Process of implementation**		
Website implementation		—

^a^No subcategory.

##### Category 4: Does the Website Achieve What It Was Designed to Achieve?

The aim of the website, as identified in the workshop analysis, was to support young people with SCIs to achieve greater health care independence, support the smooth and coordinated handover from children’s to adult health care services, and offer young people with SCIs a way to access peer support. Participants were asked to keep these aims in mind when answering the first question: “Do the website’s features achieve these outcomes? If not, why not?” All participants responded positively, and no recommendations for changes with regard to the overall outcomes were made:

I think in terms of helping to guide and support the health care transition from kids to adults, it [the website] did a pretty comprehensive job.

Jude, caregiver

##### Category 5: Website Features

Participants spoke about the features that they valued, however, had clear ideas on how the websites content could be further developed and refined.

##### 5.1: Successful Features: “That Was...Really Awesome”

On the whole, participants spoke very highly of the website:

Your website is absolutely amazing.

Morgan, caregiver

Young people and caregivers valued the wealth of information and noted that the fact sheets, resources, and links to podcasts and organizations were “handy to have in one place” (Morgan, caregiver) and “will encourage patients and their families to develop a necessary knowledge base” (Morgan, caregiver). Health care professionals affirmed this sentiment:

I suppose it’s kind of that link that I’ve always struggled with about trying to give more ownership to the young person of actually getting them to fill out bits themselves and then that kind of reveals any gaps in knowledge.

Avery, health care professional

All participants liked the simplicity of the quizzes and checklists, and their interest was piqued by the motivational videos at the start and end of the intervention:

I love the About page how it’s got your video and the Peer Support one’s got Dean...that was...really awesome...because straight away you’re like, “I want to click on that.” “I want to see what that’s about” and it’s just really kind of accessible and, teenagers, they kind of want to be fed information.

Robin, health care professional

More specifically, participants reported that the medical summary template and health care goal-planning worksheet were comprehensive and useful for everyone, even for those who had already transitioned. Furthermore, the transition checklist was “a nice way of reminding young people about their responsibilities and their self‑awareness” (Morgan, caregiver)*.*

Not only did the participants approve of the content, but they also appreciated the website’s design scheme and simplicity. This was important for young people and caregivers as the transition between health care services is both stressful and overwhelming, and they often did not have the time or patience to navigate different websites to find information:

I just want to say how nice and colourful it is and that’s also important for people. Just to make it more inviting and happier because, as you know, it might be quite overwhelming when you are going through all of that.

Morgan, caregiver

##### 5.2: Recommendations for Improvement: “One Thing I Thought Would Have Been Really Useful Is...”

The participants had valuable recommendations for the further improvement of the website. They asked for more information on the National Disability Insurance Scheme (disability support funding), more information on the general life transitions of adolescence (study, employment, and living independently), and more education on SCIs (bladder and bowel management). In addition to the medical summary template already provided, young people and caregivers also wanted a schedule of health appointments template to record all necessary regular and ongoing health appointments; for example, bladder scans and bone density tests. Young people and caregivers could then get their health care team to check that they had not missed any important health checks:

One thing I thought would have been really useful is...once you’ve got your appointments calendar lined up, to just make sure that your rehab [rehabilitation] doctor looks over it to make sure you haven’t forgotten to book in a bone scan or whatever else might be needed because it’s really hard to remember all the bits and pieces that you need to check up on and I worry that there’s something that we’re missing because no‑one’s putting it together as a whole.

Jude, caregiver

Participants also requested to hear or read more success stories and expand the website in the future to include a forum or mobile app for further peer connection opportunities:

Just add testimonials from participants with the same situation as Drew or with SCI. So it’s a good thing because you can actually build a community here. You can gain support. It’s hard. It’s hard to find support these days. So I think it’s a good thing. And also for caregivers like me.

Morgan, caregiver

It would be awesome if, in a further extension of this, that they [young people] could somehow be connected, either on the website or in a social media group or an app [application] that they could opt into.

Reese, health care professional

##### Category 6: Website Implementation

With regard to the website implementation, the researchers wanted to gain an understanding of the usability of the website as well as how best to inform young people and caregivers about it.

Although the participants acknowledged that the website needed to be functional and practical for young people of varying abilities and those that used different technologies (eg, eye gaze tracking software), they believed the website was easy to use and generally accessible to young people with multiple levels of physical abilities because of its simplicity:

All of the pages were very simple. They weren’t too overcrowded with information. So it was really easy and quick to flick through. Jessie also had a look. He was able to navigate around it quite easily and simply using his usual equipment on the computer.

Jude, caregiver

Participants appreciated the use of various formats of delivery, in particular the animation on the home page of the website, as they thought information delivered in this way was easier to digest than if a health care professional was speaking to them. In addition, participants reiterated that the simplicity of the website lent itself to being easy to use, and information was easy to comprehend for those that had limited time or a short attention span:

If anything is too complex, too busy, it just takes too much energy and it’s too exhausting to try and navigate through it and sift through what you need to know.

Morgan, caregiver

Participants believed that there was a place for all members of the SCI community to be involved in the implementation of the website, from health care professionals in the hospital setting to those in the community as well as community-based SCI support organizations. Participants believed it to be the role of all stakeholders to promote the use of the website by linking to it from their own websites and advertising it in their newsletters and social media pages. Positively, the health care professionals who worked in these roles also envisaged themselves drawing on the website in their education sessions related to transition preparation:

I would actually...Show them the basics of it and go, “Go and have a look...This is all part of your preparation.”

Avery, health care professional

I would use this, absolutely, as a resource to actually talk our patients through the process of transition and it doesn’t sound scary when it’s coming from you guys and the way that you’ve presented that information.

Avery, health care professional

#### Co-design Process Evaluation

Reflecting on the co-design process and their involvement, young people with SCIs and caregivers appreciated being given the opportunity to participate in the codevelopment of an intervention to support the HCT of young people with SCIs and caregivers. It made them feel respected, valued, and heard and provided participants with a sense of achievement:

It made us feel really respected that someone took the time to ask us what was useful for us to have in this. So often people preach at you and tell you what they think you should know and it was nice as part of this process for you to pause and ask, what would be useful for us? What did we want to see in here?

Jude, caregiver

It feels engaging. Engaging and helpful.

Drew, young person

I have a sense of achievement, it’s nice to have something you did to help other people.

Morgan, caregiver

## Discussion

### Principal Findings

Young people with SCIs and caregivers currently encounter obstacles in their HCT and report needing additional advice, information, and support to prepare for the move. This study has described the co-design and development of an HCT intervention to support the transition of young people with SCIs and caregivers from the pediatric to the adult health care setting. Information and advice to prepare for transition was purposefully presented in a manner that introduced young people to the transition process early and prompted them to learn more about their SCI and to start taking more responsibility for their own health care. Short videos informed young people about pediatric peer support services and offered “top transition tips” from a young adult who had experienced an HCT. The other tabs on the website provided links to support services, informational resources, and PDFs to support self-management skill development.

Although the purpose of this intervention was to provide support for young people with SCIs and their caregivers during their transition from pediatric to adult health care services, it must be acknowledged that this transition occurs during a broader transition process—the transition to adulthood. The transition to adulthood and its implications have been previously discussed [9,35] and, as such, this was not the focus of this study. However, in the feedback focus groups, participants reported the need for more information on the general transitions, including study, employment, and independent living. Consequently, information on where to access support on topics such as sexuality, education, and employment was added to the website, although its focus remained on supporting the move from pediatric to adult health care services.

The participatory co-design approach used in this study supported the active engagement of young people with SCIs and caregivers in the design process and resulted in the development of an intervention that addressed the current gaps in the HCT process as identified by end users. These findings support the observations of others [36,37]. For example, a study from Ireland by Coyne et al [37] reported on the co-design of a website to support the transition of young people with long-term illnesses to adult health care services. Their study highlighted that a participatory co-design approach yielded a reliable, functional, and acceptable intervention to support young people in their transition to adult health care [37]. Beaudry et al [36] similarly described a participatory co-design approach in the development of a chatbot that aimed to promote the attainment of self-care skills during the transition to adult care. They also reported that the resulting intervention was feasible for supporting engagement during HCT. Furthermore, the involvement of health care professionals in the feedback focus groups in our study ensured that we gained a broader scope for the design of the intervention, ensuring that it not only fulfilled the needs of young people with SCIs but also complemented current services.

Including end users in disability research brings knowledge and experience that may not be held by the researchers themselves and that can add to the diversity of skills and knowledge required for more appropriately designed research [38]. Furthermore, PAR and co-design principles foster empowerment as people with disabilities gain control over their lives and make decisions on matters that affect them [14]. Neither Coyne et al [37] nor Beaudry et al [36] evaluated young peoples’ experiences of being involved in the co-design process; however, our study did. Evaluation data from young people on their involvement in the co-design process highlighted that their inclusion empowered them, gave them a voice, and provided them with an opportunity to contribute to an intervention that would make a difference in their lives and the lives of others. This provides further evidence of the importance of giving young people with disabilities the opportunity to authentically and meaningfully participate in the research and codevelopment of interventions that affect their lives.

### Strengths and Limitations

Evaluation of the co-design process indicated that the participants valued the opportunity to be part of the development of a solution and appreciated being given a voice. However, the iterative and cyclical nature of the co-design process did present some challenges. Recruitment for the study and maintenance of engagement was a challenge across the different phases of the study. Of the 9 young people and caregivers who participated in the interviews during phase 1 of the study, 8 (89%) returned to participate in the co-design workshops, and 4 (44%) participated in the focus groups in phase 2. Reasons for the dropout included family stressors, relocation to another country, and nonresponse to phone or email. Owing to a paucity of participant numbers, we did not achieve a representative research sample, with no LGBTQ+ and no cultural and linguistically diverse representation (including Aboriginal and Torres Strait Islander input). A further limitation of our study relates to the inability to compare our findings with other similar studies because of a paucity of written literature on this topic [16].

Owing to the COVID-19 pandemic, activities initially intended to be held in person were moved to the web. This modification had its advantages as it eliminated geographical and mobility barriers to participation and fostered inclusive research practices. However, because of the additional pressures of COVID-19 and homeschooling on families, it was difficult to find times that were suitable to all, and it required a substantial amount of preparatory work on the part of the principal researcher (EAB) to organize workshops and focus groups.

The next phase of the PAR study is to assess the acceptability and feasibility of the HCT intervention. We plan to roll out the website with the same participants and conduct short evaluation telephone interviews based on the 8 focus areas in the framework by Bowen et al [39].

### Conclusions

Engaging young people with SCIs and caregivers in the co-design of an HCT intervention has produced, in a relatively short time frame, a great depth of insight into the transition needs of young people with SCIs and their priorities for support. The result has been the collaborative development of an intervention that young people with SCIs, caregivers, and health care professionals believe will support the transition from child to adult health care services and equip young people with SCIs with practical and helpful tools to take charge of their health care. This is the first study to co-design and develop an HCT intervention in partnership with young people with SCIs and caregivers. Although the study sample was small, it has shown that it is possible to meaningfully engage young people with SCIs and caregivers in the co-design of an HCT intervention that leads to enhanced end-user acceptability.

## Appendix

Multimedia Appendix 1 Project phase and objective alignment with the Care Transitions Framework domains. It displays how the phases of the project and study objectives align with the Care Transitions Framework domains.

Multimedia Appendix 2 Young person co-design workshop participant booklet (activity booklet sent to young people in preparation for the co-design workshop).

Multimedia Appendix 3 Caregiver co-design workshop participant booklet (activity booklet sent to young people in preparation for the co-design workshop).

Multimedia Appendix 4 Screenshots of the icebreaker used across both co-design workshops. This image presents 2 screenshots of the randomized question spin wheel used as an icebreaker in the co-design workshops.

Multimedia Appendix 5 Screenshots of the 3-stage process from activity 2 of the young person workshop. This figure shows young people’s responses to the 3 questions posed in the co-design activity.

Multimedia Appendix 6 Screenshots of the 3-stage process of the caregiver workshop. This image shows caregivers’ responses to the 3 questions posed in the co-design activity.

Multimedia Appendix 7 Examples of meaning units, condensed meaning units, codes, subcategories, and categories (an example of the how the data were condensed into meaning units, coded, and categorized).

Multimedia Appendix 8 Young person workshop evaluation (this image displays young people’s responses to the workshop evaluation question).

## Abbreviations

HCT: health care transition
NSW: New South Wales
PAR: participatory action research
SCI: spinal cord injury

## References

[1] Mulcahey MJ, Vogel LC, Sheikh M, Arango-Lasprilla JC, Augutis M, Garner E, Hagen EM, Jakeman LB, Kelly E, Martin R, Odenkirchen J, Scheel-Sailer A, Schottler J, Taylor H, Thielen CC, Zebracki K (2017). Recommendations for the National Institute for Neurologic Disorders and Stroke spinal cord injury common data elements for children and youth with SCI. Spinal Cord.

[2] Schmidt A, Ilango SM, McManus MA, Rogers KK, White PH (2020). Outcomes of pediatric to adult health care transition interventions: an updated systematic review. J Pediatr Nurs.

[3] (2014). Key Principles for Transition of Young People from Paediatric to Adult Health Care. Agency for Clinical Innovation and Trapeze, The Sydney Children’s Hospitals Network.

[4] Blum RW, Garell D, Hodgman CH, Jorissen TW, Okinow NA, Orr DP, Slap GB (1993). Transition from child-centered to adult health-care systems for adolescents with chronic conditions. A position paper of the Society for Adolescent Medicine. J Adolesc Health.

[5] Stewart D (2009). Transition to adult services for young people with disabilities: current evidence to guide future research. Dev Med Child Neurol.

[6] Gray WN, Schaefer MR, Resmini-Rawlinson A, Wagoner ST (2018). Barriers to transition from pediatric to adult care: a systematic review. J Pediatr Psychol.

[7] White PH, Cooley WC, Transitions Clinical Report Authoring Group, American Academy of Pediatrics, American Academy of Family Physicians, American College of Physicians (2018). Supporting the health care transition from adolescence to adulthood in the medical home. Pediatrics.

[8] Acuña Mora M, Saarijärvi M, Moons P, Sparud-Lundin C, Bratt EL, Goossens E (2019). The scope of research on transfer and transition in young persons with chronic conditions. J Adolesc Health.

[9] Porto A, Anderson L, Kalinich T, Deane KC, Vogel LC, Zebracki K (2020). Understanding transition for youth with spinal cord injury: youth and caregiver perceptions. J Spinal Cord Med.

[10] Baker I, de Paula A, Serratore L, Hanna M, Diviney K, Clark N, Bailey V (2010). Towards independence: the New South Wales (Australia) experience of transition to adulthood of young people with spinal cord injury. Top Spinal Cord Inj Rehabil.

[11] Steen M, Manschot M, De Koning N (2011). Benefits of co-design in service design projects. Int J Des.

[12] Thabrew H, Fleming T, Hetrick S, Merry S (2018). Co-design of eHealth interventions with children and young people. Front Psychiatry.

[13] Baum F, MacDougall C, Smith D (2006). Participatory action research. J Epidemiol Community Health.

[14] Balcazar F, Keys C, Kaplan MA, Suarez-Balcazar Y (1998). Participatory action research and people with disabilities: principles and challenges. Can J Rehabil.

[15] Kelly PJ (2005). Practical suggestions for community interventions using participatory action research. Public Health Nurs.

[16] Bray EA, Everett B, George A, Salamonson Y, Ramjan LM (2021). Co-designed healthcare transition interventions for adolescents and young adults with chronic conditions: a scoping review. Disabil Rehabil.

[17] (2020). Evidence and utilisation of spinal cord injury services in NSW: Evidence Series. Agency for Clinical Innovation and Government of New South Wales.

[18] Bray EA, George A, Everett B, Salamonson Y, Ramjan L (2021). Protocol for developing a healthcare transition intervention for young people with spinal cord injuries using a participatory action research approach. BMJ Open.

[19] Rojas SL, Ashok M, Morss Dy S, Wines RC, Teixeira-Poit S (2014). Contextual Frameworks for Research on the Implementation of Complex System Interventions.

[20] O'Brien BC, Harris IB, Beckman TJ, Reed DA, Cook DA (2014). Standards for reporting qualitative research: a synthesis of recommendations. Acad Med.

[21] New PW, Baxter D, Farry A, Noonan VK (2015). Estimating the incidence and prevalence of traumatic spinal cord injury in Australia. Arch Phys Med Rehabil.

[22] New PW, Farry A, Baxter D, Noonan VK (2013). Prevalence of non-traumatic spinal cord injury in Victoria, Australia. Spinal Cord.

[23] Vogel LC, Betz RR, Mulcahey MJ, Zebracki K, Kirshblum S, Lin VW (2017). Spinal cord injuries and disorders in children and adolescents. Spinal Cord Medicine. 3rd edition.

[24] Choi EK, Bae E, Jang M (2021). Transition programs for adolescents and young adults with spina bifida: a mixed-methods systematic review. J Adv Nurs.

[25] Holmbeck GN, Kritikos TK, Stern A, Ridosh M, Friedman CV (2021). The transition to adult health care in youth with spina bifida: theory, measurement, and interventions. J Nurs Scholarsh.

[26] Elo S, Kyngäs H (2008). The qualitative content analysis process. J Adv Nurs.

[27] Erlingsson C, Brysiewicz P (2017). A hands-on guide to doing content analysis. Afr J Emerg Med.

[28] Ponterotto JG (2006). Brief note on the origins, evolution, and meaning of the qualitative research concept thick description. Qual Rep.

[29] Shenton AK (2004). Strategies for ensuring trustworthiness in qualitative research projects. Educ Inf.

[30] Olmos-Vega FM, Stalmeijer RE, Varpio L, Kahlke R (2022). A practical guide to reflexivity in qualitative research: AMEE Guide No. 149. Med Teach.

[31] Alminde S, Warming H (2019). Future workshops as a means to democratic, inclusive and empowering research with children, young people and others. Qual Res.

[32] Müllert N, Jungk R (1987). Future Workshops: How to Create Desirable Futures.

[33] Bengtsson M (2016). How to plan and perform a qualitative study using content analysis. NursingPlus Open.

[34] Bray EA (2022). SCI Healthcare Transition.

[35] Zebracki K, Anderson C, Chlan K, Vogel L (2010). Outcomes of adults with pediatric-onset spinal cord injury: longitudinal findings and implications on transition to adulthood. Top Spinal Cord Inj Rehabil.

[36] Beaudry J, Consigli A, Clark C, Robinson KJ (2019). Getting ready for adult healthcare: designing a chatbot to coach adolescents with special health needs through the transitions of care. J Pediatr Nurs.

[37] Coyne I, Prizeman G, Sheehan A, Malone H, While AE (2016). An e-health intervention to support the transition of young people with long-term illnesses to adult healthcare services: design and early use. Patient Educ Couns.

[38] Joss N, Cooklin A, Oldenburg B (2016). A scoping review of end user involvement in disability research. Disabil Health J.

[39] Bowen DJ, Kreuter M, Spring B, Cofta-Woerpel L, Linnan L, Weiner D, Bakken S, Kaplan CP, Squiers L, Fabrizio C, Fernandez M (2009). How we design feasibility studies. Am J Prev Med.

