# Supplementary material for: Developing a Health Care Transition Intervention With Young People With Spinal Cord Injuries: Co-design Approach
Source: JMIR Form Res. 2022 Jul 28;6(7):e38616. doi: 10.2196/38616 (PMC9377469; doi:10.2196/38616)
Supplement: Multimedia Appendix 1 [file formative_v6i7e38616_app1.pdf]

Multimedia Appendix 1. Project phase and objective alignment with the Care Transitions Framework Domains

Phase	Study Objectives	Care Transitions Framework Domain	
**Phase 1 - PLAN**<br>*Exploring current experiences and unmet needs*<br>o Scoping review<br>o Consultations with services and stakeholders<br>o Semi-structured individual interviews<br>**Completed**	• Identify current services and resources that aid in facilitating the transition of young people with SCI from paediatric to adult health services.<br>• Understand the experience of transition for young people with SCI and their parents/caregivers.<br>• Explore the current needs of young people with SCI to identify gaps within the transition process.	Characteristics and Roles of Patients and Caregivers	Define the characteristics and roles of the patient and caregiver<br>• What patient and caregiver characteristics will impact their ability to engage in the intervention or benefit from it?
**Phase 2 - ACT**<br>*Co-designing the HCT intervention*<br>o Co-design workshops<br>o Development of prototype HCT intervention<br>o Feedback focus groups<br>**Reported**	• Explore the current needs of young people with SCI to identify gaps within the transition process.<br>• Co-design and develop a HCT intervention to support young people with SCI.	Intervention Characteristics	Define the intervention<br>• What is the intervention designed to achieve?<br>• What are the features of the intervention?<br>• Who is the intended target group?
		Process of Implementation	Define the process required to achieve desired level of use<br>• What processes will be applied to achieve individual and organisational level use of the intervention?<br>• What roles will providers, teams, patients, and caregivers carry out?
**Phase 3 - REVIEW**<br>*Implementing and evaluating the HCT intervention*<br>o Refinement of HCT intervention<br>o HCT intervention evaluation<br>**In progress**	• Implement the HCT intervention and evaluate its acceptability and feasibility in supporting the transition process.	Measures of Implementation	Define successful implementation<br>• What attributes of the implementation process demonstrate it was carried out well and can be replicated, scaled and sustained?
		Outcomes	Define the outcomes<br>• What specific, measurable outcomes will result from the intervention?

Legend: Multimedia Appendix 1 displays how the phases of the project and study objectives align with the Care Transitions Framework domains.
