# Supplementary material for: Developing a Health Care Transition Intervention With Young People With Spinal Cord Injuries: Co-design Approach
Source: JMIR Form Res. 2022 Jul 28;6(7):e38616. doi: 10.2196/38616 (PMC9377469; doi:10.2196/38616)
Supplement: Multimedia Appendix 2 [file formative_v6i7e38616_app2.pdf]

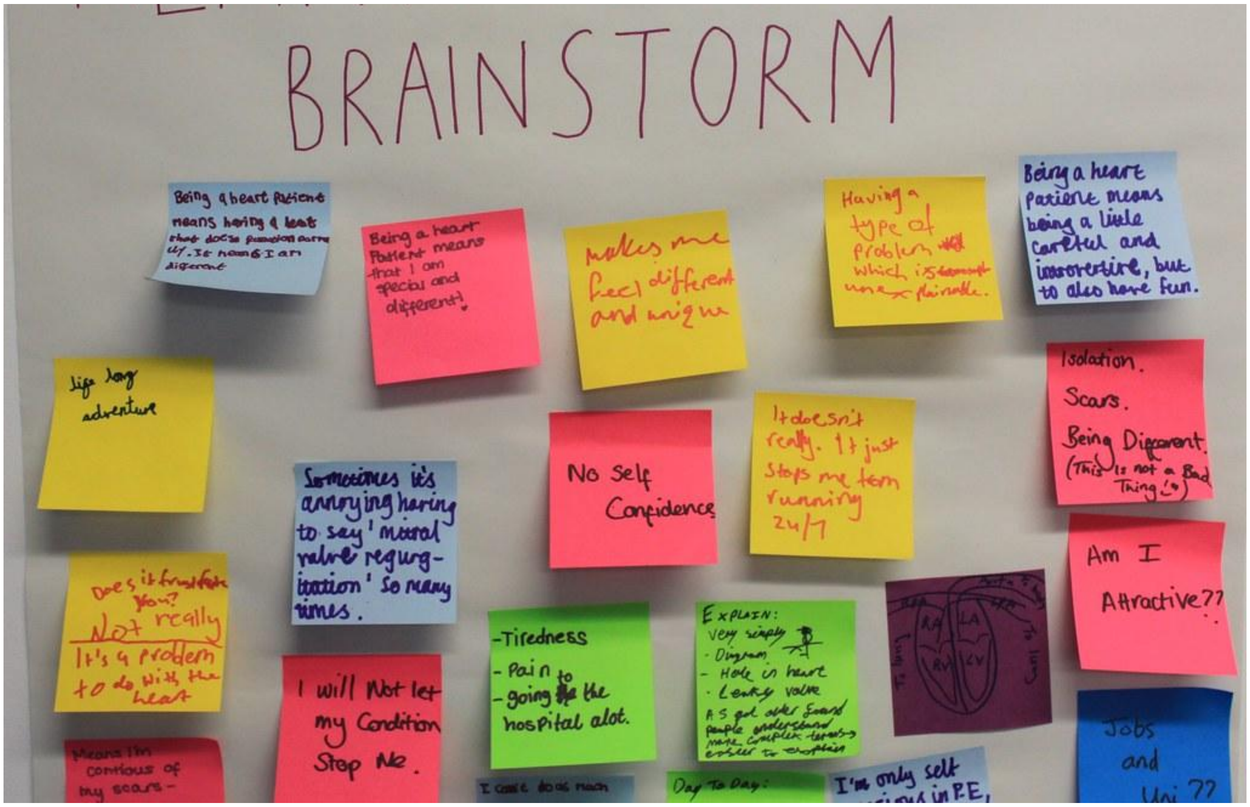

Supporting Young People with SCI Moving from Paediatric to Adult Healthcare Services

Young persons co-design workshop

23.08.2021

—

Western Sydney University

Overview

By now you will have taken part in an interview that explored your experiences, needs, and expectations regarding the move from the children's hospital to the adult hospital and the transfer to other adult health services. In the upcoming workshop we will be reviewing the common themes from your interviews and working together to design a tool, resource or program that will help others in their transition.

Goals for the workshop

1. Review and discuss the common themes and thoughts from the interviews
2. Design a tool, resource or program that will help others in their move from children to adult healthcare services.

Pre-workshop

In preparation for this workshop I would like you to complete the following activities. We will discuss your thoughts in more detail when we meet as a group.

Activity one

Please read through the following words and phrases from the interviews. Can they be grouped into themes that address an experience, need or expectation?

Please use the boxes provided to group together the words or phrases that represent the concepts of expectations for the move, experiences of the move and what is needed to make the move better.

You can do this by copying and pasting them into the table provided.

Some words or phrases may be used in more than one box and can be both positive or negative.

There are no wrong or right answers and you will get a chance to explain your reasoning in the workshop.

Scared

Support and advice from someone who has been through the transition already

Opportunities to meet new doctors

Information on new doctors, what they do and how to contact them

Information about health services in the community

Support at first appointments in adult hospital

Support when I need it

One support person I know I can call

Start talking about transition earlier

Information on what happens when things go wrong

Nervous

Information on how the adult hospital works

Summary that I can give to new doctors (medication, medical history, events of injury)

Difficult to communicate needs and questions

Help to collect medical information

Information on services that help with transition

Excited

Customisable / individualised information

Opportunity to meet others with a SCI

No clear transition process

Activities for young people staying in adult hospital

Support to develop self management skills/independence

Familiar

Adolescent support service (16 - 21/22 years)

Opportunities to try new things

One place for all information

Team approach

Handover meeting with both paediatric and adult teams

Role play and practice to talk to doctors

Lists of questions to ask doctors

No communication between child and adult services

Uncertainty

More choice and control

Education for new doctors

More planning around move

Guide on adolescent life transitions

Holistic (whole person) approach to healthcare

More correspondence outlining the transition process

Independence

Comfortable

Overwhelming

Sudden change

More communication between paediatric and adult setting

Specific meetings on transition

Slower pace for transitioning

Schedule for check ups in adult hospital

<table>
<tr><td rowspan="4">Mindsets
What words, phrases or ideas describe the feelings people experience before during or after transition?</td><td>For example: Scared</td></tr>
<tr><td>Before:</td></tr>
<tr><td>During:</td></tr>
<tr><td>After:</td></tr>
<tr><td>Experiences
What words, phrases or ideas represent an experience with the move from children’s to adult healthcare services?</td><td>For example: Sudden change</td></tr>
<tr><td>Transition needs or recommendations
What words, phrases or ideas describe gaps or recommendations for the transition process?</td><td>For example: Group peer support</td></tr>
</table>

Activity Two

Please think about the following questions and brainstorm some ideas to discuss in the workshop. You can use the space provided to write down your thoughts or feel free to tap into your creative side and draw your ideas, write a poem, design a collage, take photos, it's up to you! Bring these along to the workshop as a prop to talk through your ideas.

What is the change you want to see in your move from children's to adult healthcare?

In a perfect world how can this be achieved?

How can we make this possible today? What are your thoughts on a practical solution that may help you or others with the move?

Workshop

I am really excited to have you involved and look forward to working with you on the day.

Below is an outline of what will happen on the day of the workshop. See you there!

**Timing**	
0:00-0:05<br>(5 mins)	Welcome and orientation
0:05-0:15<br>(10 mins)	Ice breaker
0:15-0:45<br>(20-30 mins)	**Activity one**<br>Review and discuss the common themes and thoughts from the interviews
0:45-0:50<br>(5 mins)	Break
0:50-1:30<br>(40 mins)	**Activity Two**<br>Co-designing the tool, program or resource for supporting young people with SCI moving from childrens to adult healthcare
1:30-1:35<br>(5 mins)	Closing reflections<br>Questions
