# Supplementary material for: Developing a Health Care Transition Intervention With Young People With Spinal Cord Injuries: Co-design Approach
Source: JMIR Form Res. 2022 Jul 28;6(7):e38616. doi: 10.2196/38616 (PMC9377469; doi:10.2196/38616)
Supplement: Multimedia Appendix 3 [file formative_v6i7e38616_app3.pdf]

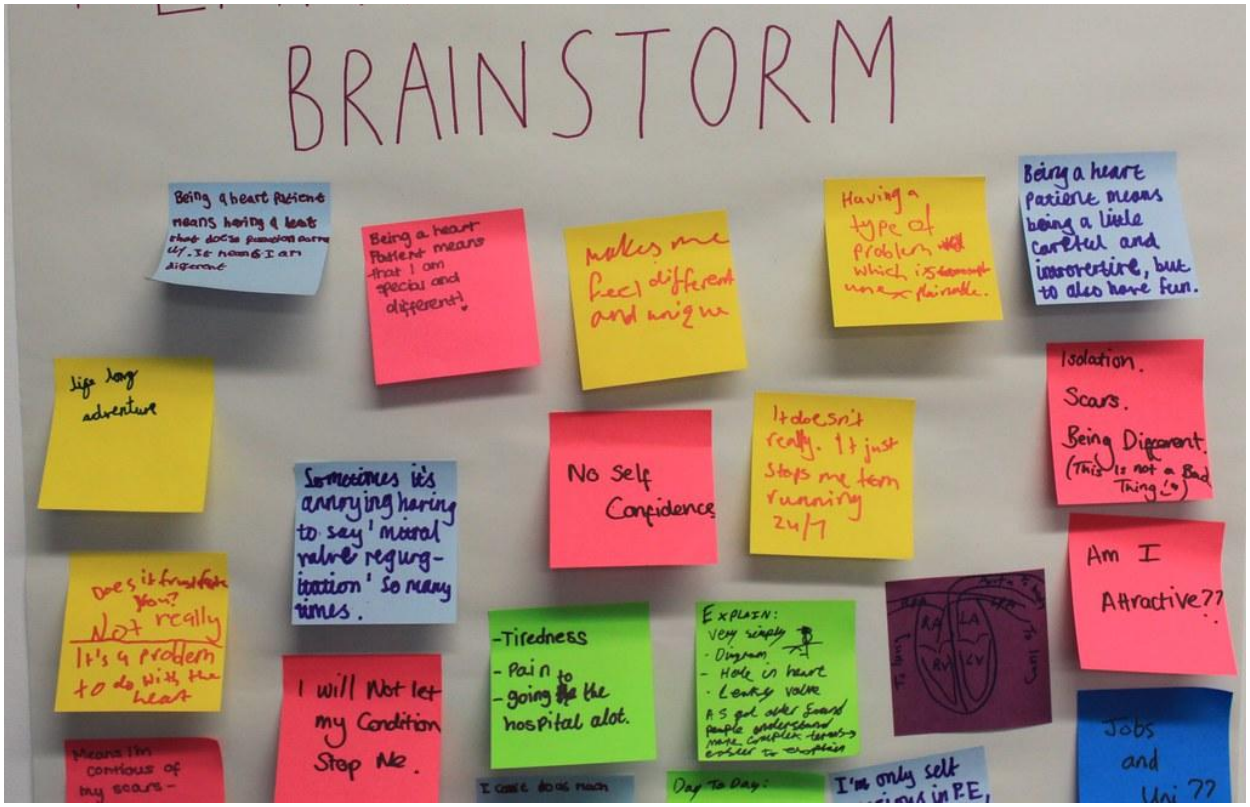

Supporting Young People with SCI Moving from Paediatric to Adult Healthcare Services

Parents/caregivers co-design workshop

01.09.2021

—

Western Sydney University

Overview

By now you will have taken part in an interview that explored you and your childs experiences, needs, and expectations regarding the move from the children's hospital to the adult hospital and the transfer to other adult health services. In the upcoming workshop we will be reviewing the common themes from your interviews and working together to design a tool, resource or program that will help others in their transition.

Goals for the workshop

1. Review and discuss the common themes and thoughts from the interviews
2. Design a tool, resource or program that will help other young people in their move from children to adult healthcare services.

Pre-workshop

In preparation for this workshop I would like you to review the following questions. We will discuss your thoughts in more detail when we meet as a group.

What is the change you want to see in your childs move fom children's to adult healthcare?

In an perfect world how can this be achieved?

How can we make this possible today? What are your thoughts on a practical solution that may help you or others with the move?
