# Supplementary material for: Developing a Health Care Transition Intervention With Young People With Spinal Cord Injuries: Co-design Approach
Source: JMIR Form Res. 2022 Jul 28;6(7):e38616. doi: 10.2196/38616 (PMC9377469; doi:10.2196/38616)
Supplement: Multimedia Appendix 4 [file formative_v6i7e38616_app4.pdf]

Multimedia Appendix 4: Screenshots of the icebreaker used across both co-design workshops

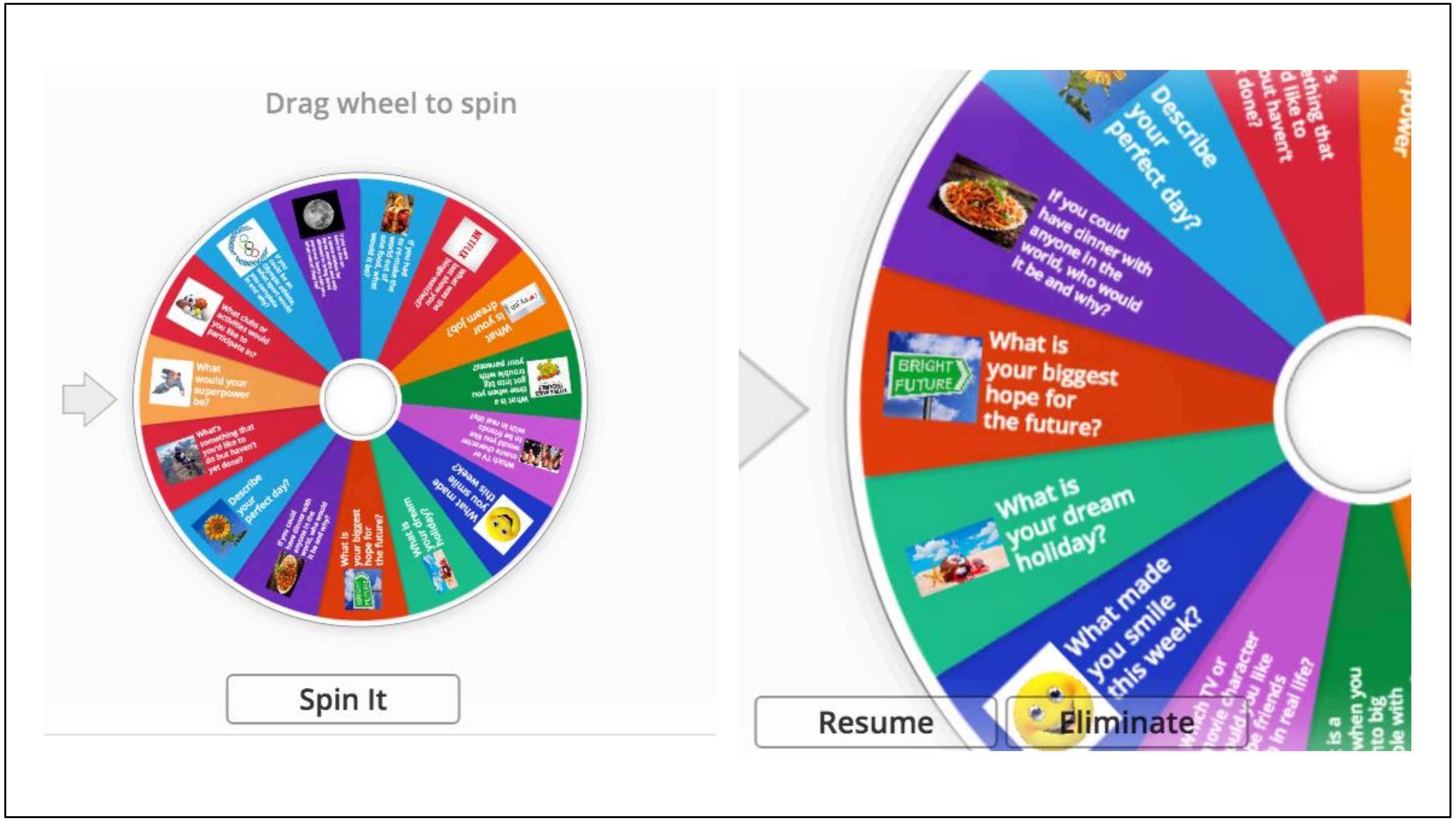

Legend: This image presents two screenshots of the icebreaker used in the co-design workshops. It shows a question generating spin wheel.
