# Supplementary material for: Developing a Health Care Transition Intervention With Young People With Spinal Cord Injuries: Co-design Approach
Source: JMIR Form Res. 2022 Jul 28;6(7):e38616. doi: 10.2196/38616 (PMC9377469; doi:10.2196/38616)
Supplement: Multimedia Appendix 5 [file formative_v6i7e38616_app5.pdf]

Multimedia Appendix 5: Screenshots of 3-stage process from activity two of the young person workshop

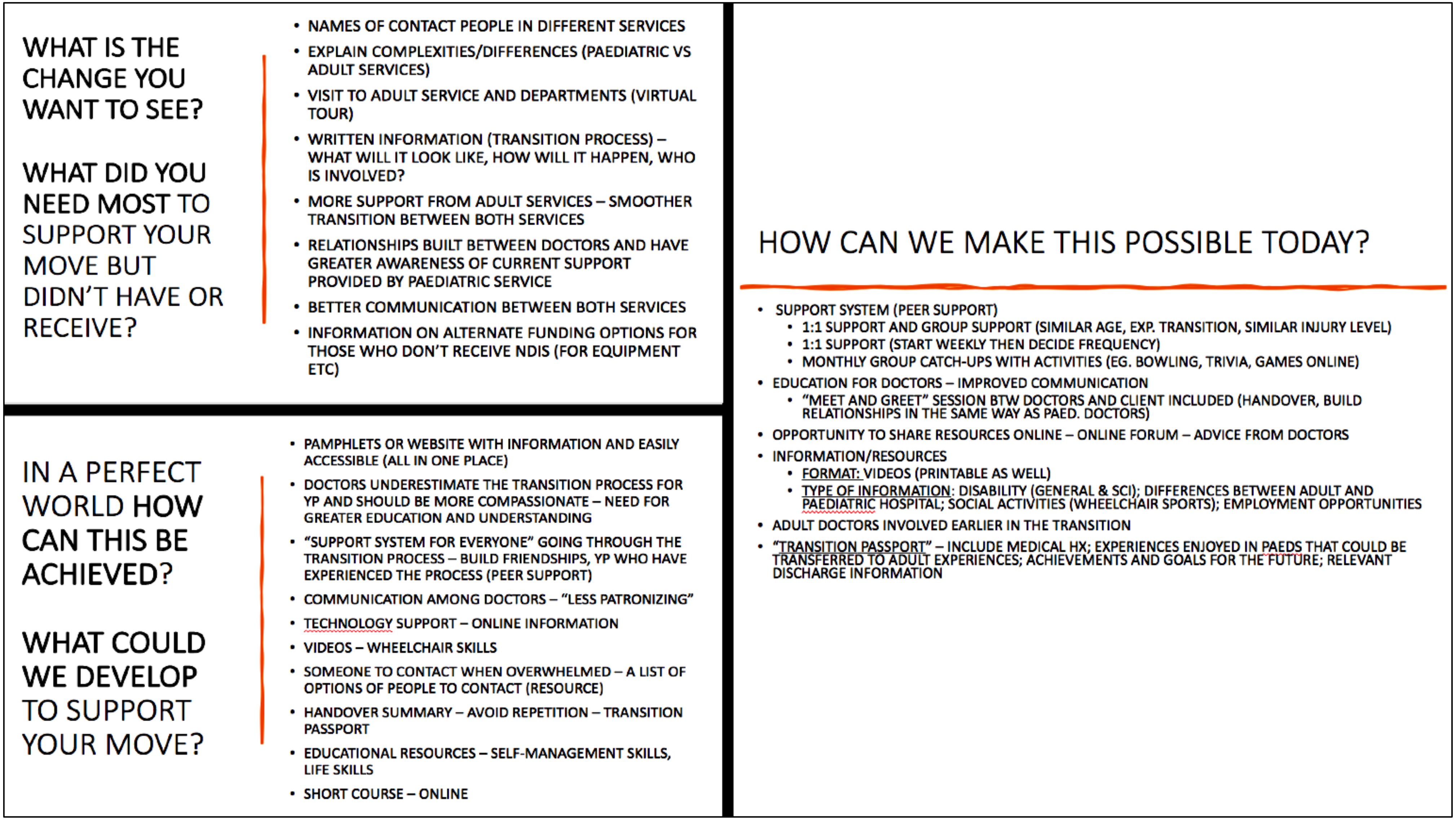

Legend: This figure shows young people's responses to the three questions posed in the co-design activity.
