# Supplementary material for: Developing a Health Care Transition Intervention With Young People With Spinal Cord Injuries: Co-design Approach
Source: JMIR Form Res. 2022 Jul 28;6(7):e38616. doi: 10.2196/38616 (PMC9377469; doi:10.2196/38616)
Supplement: Multimedia Appendix 7 [file formative_v6i7e38616_app7.pdf]

Multimedia Appendix 7. Examples of meaning units, condensed meaning units, codes, subcategories and categories

**Meaning unit**	**Condensed meaning unit**	**Code**	**Subcategory**	**Category**
"There just needs to be more support during the transition process by the doctors from the adult hospital and there needs to be a transition support service so, yeah, adolescents don't just jump straight into adult services so that they can feel more comfortable and supported."	*"There just needs to be more support during the transition process by the doctors from the adult hospital"*	More support from healthcare professionals	Coordinated handover between services: *"For there to be more of a relationship built with the doctor before the transition occurs"*	What is the intervention designed to achieve?

Legend: Multimedia Appendix 7 provides an example of the how the data was condensed into meaning units, coded and categorised.
