# Supplementary material for: Developing a Health Care Transition Intervention With Young People With Spinal Cord Injuries: Co-design Approach
Source: JMIR Form Res. 2022 Jul 28;6(7):e38616. doi: 10.2196/38616 (PMC9377469; doi:10.2196/38616)
Supplement: Multimedia Appendix 8 [file formative_v6i7e38616_app8.pdf]

Multimedia Appendix 8: Young person's workshop evaluation

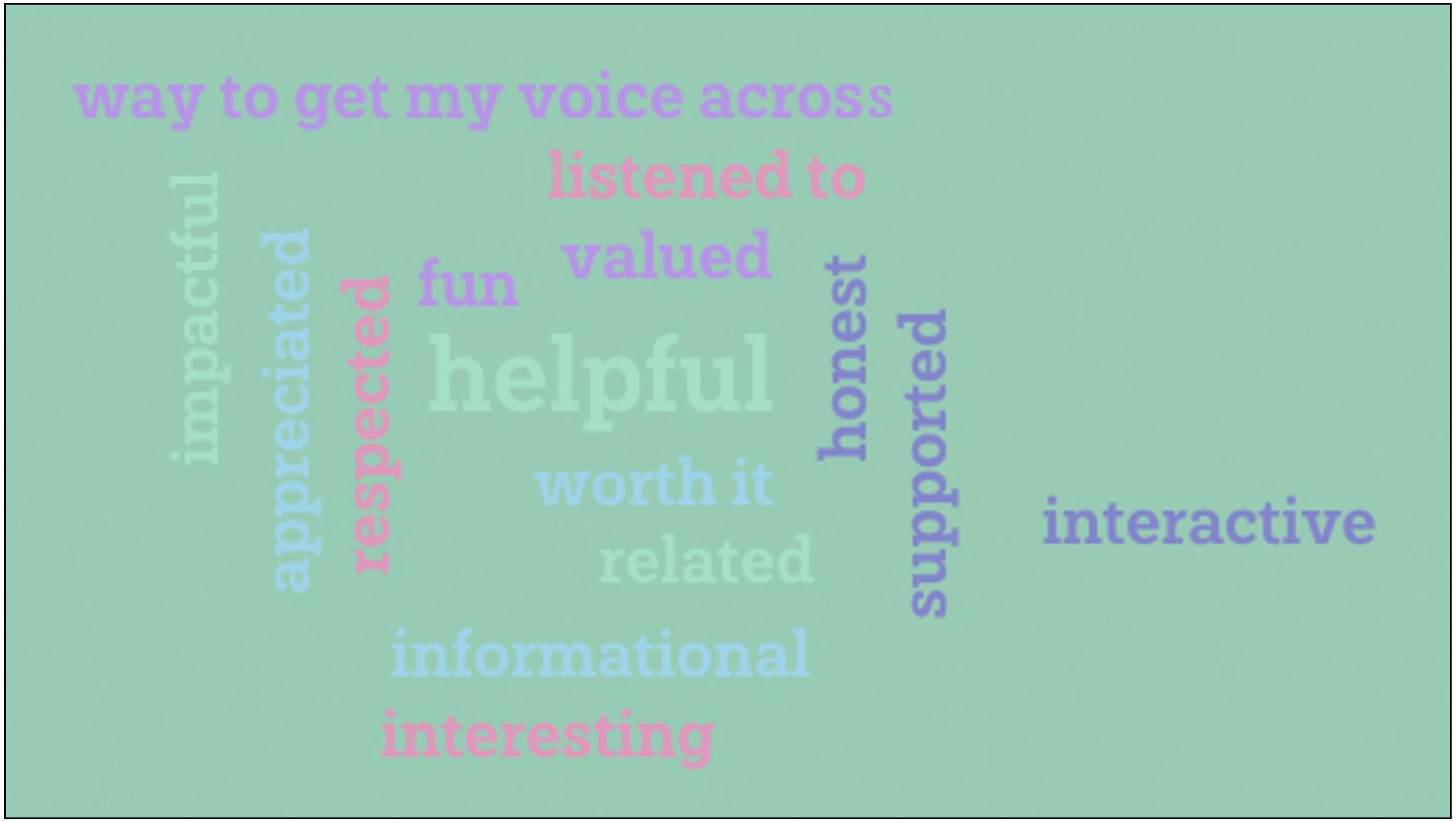

Legend: This image displays young people's responses to the workshop evaluation question.
